# Case Report: Rapid Progression of Cognitive Dysfunction as an Initial Feature of Systemic Lupus Erythematosus With Leukoencephalopathy: A Case Report and Literature Review

**DOI:** 10.3389/fneur.2022.934335

**Published:** 2022-07-11

**Authors:** Yukun Feng, Teng Yu, Qin Xiao, Xiaodong Yang

**Affiliations:** ^1^Department of Neurology and Institute of Neurology, Ruijin Hospital, Shanghai Jiao Tong University School of Medicine, Shanghai, China; ^2^Department of Neurology, Hainan General Hospital, Hainan Affiliated Hospital of Hainan Medical University, Haikou, China; ^3^Department of Pathology, Ruijin Hospital, Shanghai Jiao Tong University School of Medicine, Shanghai, China

**Keywords:** neuropsychiatric systemic lupus erythematosus (NPSLE), leukoencephalopathy syndrome, rapidly progressive dementia (RPD), diffusion-weighted image (DWI), MRI

## Abstract

Neuropsychiatric systemic lupus erythematosus (NPSLE) has been considered to have high morbidity and mortality. Thus, earlier recognition and treatment are of great importance. However, the rapid progression of cognitive dysfunction with leukoencephalopathy as an initial presentation in SLE is rarely described. We report a case in which an elderly man experienced rapidly progressive cognitive impairment with bilateral, symmetric, and diffuse leukoencephalopathy with lasting diffusion-weighted image hyperintensity. An immunological workup showed low complement levels and positivity for antinuclear antibody -speckle and Coombs tests in the patient's serum samples. He had an appropriate improvement in cognitive function after receiving a combination of various immunotherapies. Long-term follow-up showed clinical improvement, including rheumatological labs and neuroimaging. A review of the literature on NPSLE with leukoencephalopathy and a summary of all reported cases to date are also presented. Our case indicated that isolated leukoencephalopathy in NPSLE, as an indicator of severe NPSLE, can be recognized early. Immunotherapy is warranted given the possibility of clinical improvement.

## Introduction

Systemic lupus erythematosus (SLE) is a chronic inflammatory autoimmune disorder characterized by multiorgan involvement, the vast majority of whom are females of childbearing age (1). It presents clinically heterogeneous, usually involving in renal, dermatological, neuropsychiatric, musculo-skeletal and cardiovascular symptoms (1). Approximately 50 to 80% of patients have neuropsychiatric involvement in SLE (NPSLE) (2). NPSLE has been associated with an increase in the mortality rate and a lower quality of life (3, 4). Neuropsychiatric involvement occurs in the early stage of SLE and presents a variety of symptoms (4, 5). These include psychiatric manifestations (mood disorders, cognitive impairment, and psychosis), stroke, seizures, myelopathy, chorea, and headaches (5). While neuropsychiatric events as initial symptoms in SLE patients are rare (4). Moreover, neuropsychiatric manifestations were classified into focal and diffuse neuropsychological syndromes (6). In the diffuse neuropsychological syndromes, isolated diffuse leukoencephalopathy with acute confusional state or cognitive dysfunction in lupus has rarely been reported and presents benign or malignant outcomes (7). Given the heterogeneity of the clinical manifestations, it is extremely difficult to recognize and treat early.

Here, our case report illustrates neuropsychiatric symptoms as an initial and only manifestation of SLE, with bilateral, symmetric, and diffuse leukoencephalopathy with lasting hyperintense diffusion-weighted images (DWI). We reviewed the literature on the clinical characteristics of and treatment responses for this condition. These characteristics are rare and make diagnosis challenging. Such a presentation represents a severe variant of NPSLE requiring aggressive immunosuppressive therapies.

## Methods and Results

### Patient Data

A 58-year-old male initially presented to our hospital with cognitive dysfunction and gait disturbance. He started to have memory decline, impaired attention, and deficits in processing speed 2 months prior to his admission. Subsequently, he presented dysphagia and aphasia. His movement gradually became sluggish and even led to him being bedridden long-term. Meanwhile, he had never suffered from the malar, or butterfly rash, glomerulonephritis, arthralgias, and anemia etc.

The serological workup of immunology (Table 1) showed an increase in the erythrocyte sedimentation rate (ESR) ( 63 mm/h), anti-cardiolipin antibody IgM (42.9 MPL) level and C-reactive protein (CRP) (17 mg/L) level, a homogeneous antinuclear antibody-speckle (ANA) 320X test, Coombs test and anti-ribosomal P positivity and a decrease in C3 complement (C3) (0.60 g/L), C4 complement (C4) (0.06 g/L) and total completement activity (CH50) (1.0 U/mL) levels. Cerebral spinal fluid (CSF) analysis (Table 1) was negative under normal pressure, including for paraneoplastic antibodies (hu, ri, CV2, ANNA-3, PCA-2, YO, MA2, Amphiphysin, Tr, GAD) and autoimmunity encephalitis-associated antibodies (NMDA, AMPA1, AMPA2, IgLON5, GABA, LGI, CASPR2, MBP, MOG, AQP4). The leukodystrophy-associated gene panel testing results and exercise testing on serum lactate were negative.

**Table 1:** Lab results and normal values.

**Lab test**	**Patient value**	**Patient value**	**Patient value**	**Normal value**
	**(1 month)**	**(3 months)**	**(12 months)**	
CRP	17 mg/L	10 mg/L	1 mg/L	<10 mg/L
ESR	63 mm/h	6 mm/h	5 mm/h	Male: 0-15 mm/h
Complements (C3, C4)	C3: 0.60 g/L	0.89 g/L	1.08 g/L	0.74–1.4 g/L
	C4: 0.06 g/L	0.35 g/L	0.34 g/L	0.1-0.4 g/L
Total completement activity (CH50)	1.0 U/mL	42.0 U/mL	44 U/mL	23.0-46 U/mL
β2-glycoprotein IgA	≤ 9.4 SAU	–	≤ 9.4 SAU	<20 SAU
β2-glycoprotein IgG	≤ 9.4 SGU	–	≤ 9.4 SGU	<20 SGU
β2-glycoprotein IgM	12.7 SMU	–	≤ 9.4 SMU	<20 SMU
Anti-cardiolipin antibody IgG	9.4 GPL	–	≤ 9.4 GPL	<16 GPL (Negative)
Anti-cardiolipin antibody IgM	42.9 MPL	–	≤ 9.4 MPL	>20 MPL (Positive)
Lupus anticoagulant	1.03	–	1.01	<1.2 (Negative)
ANA	0.2639	–	Negative	Negative, <1:80
Anti-dsDNA lgG	126.8 IU/mL	–	29.4 U/mL	<200 IU/mL (Negative)
Anti-Smith antibody	Negative	–	Negative	Negative
Anti-SSA antibody	Negative	–	Negative	Negative
Anti-SSB antibody	Negative	–	Negative	Negative
Anti-RNP antibody	Negative	–	Negative	Negative
Anti-Smith/RNP antibody	Negative	–	Negative	Negative
Anti-ribosomal P antibody	Positive	–	Negative	Negative
CSF chemistry		–		
Protein	436.17 mg/L	–	–	150-450 mg/L
Glucose	3.27 mmol/L	–	–	2.5-4.4 mmol/L
CSF cytology	1.00 ×10^6^/L	–	–	0-5.00 ×10^6^/L
CSF microbial sequencing	Negative	–	–	Negative
Oligoclonal banding	Negative	–	–	Negative

**Figure 1:**
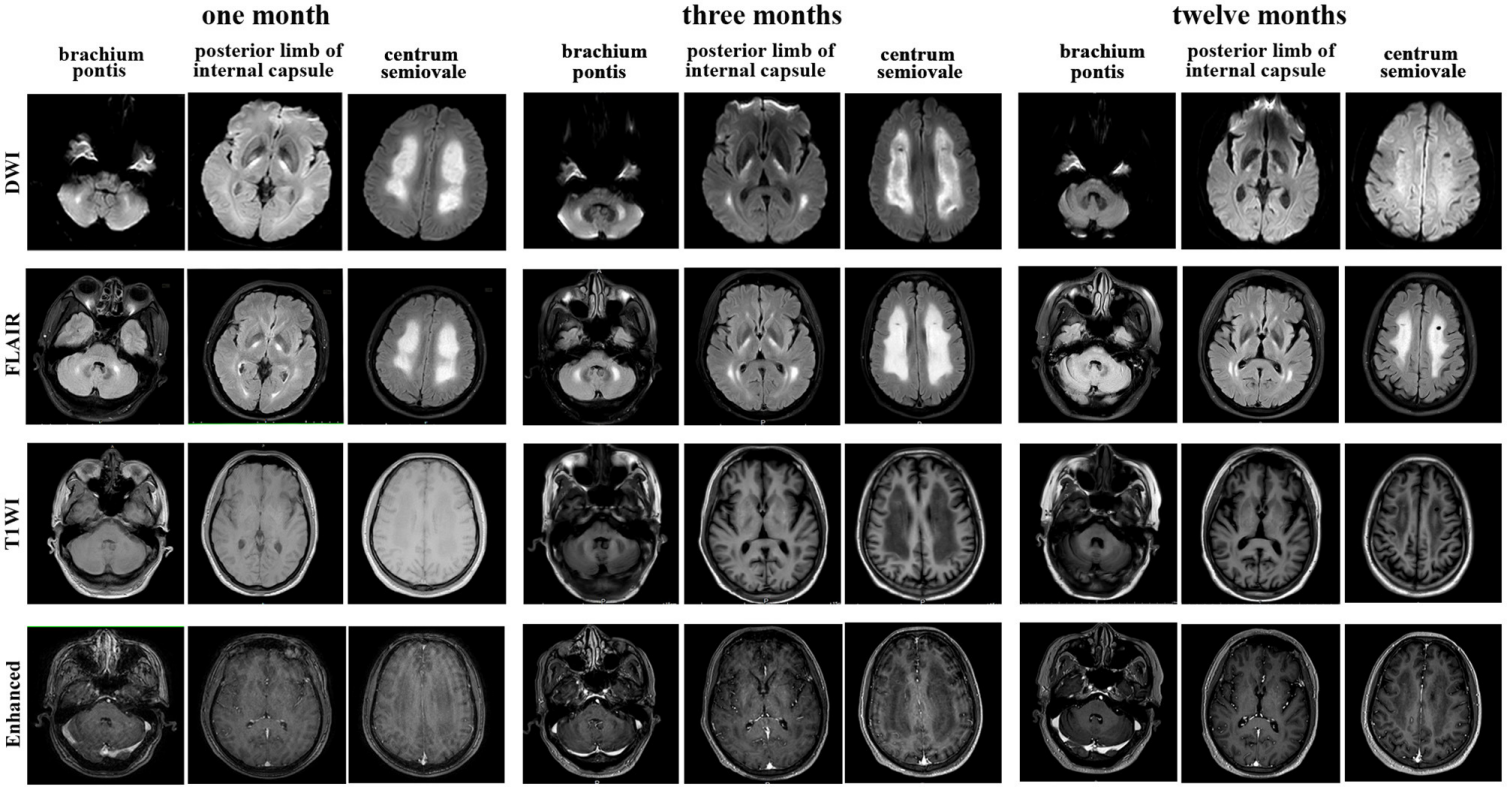
Brain magnetic resonance imaging (MRI) performed at the 1-month, 3-month, and 12-month follow-ups showed T1-weighted, diffusion-weighted images (DWI), fluid-attenuated inversion recovery (FLAIR) and enhanced T1 MRI axial images of the centrum semiovale, brachium pontis and internal capsule lesions.

**Figure 2:**
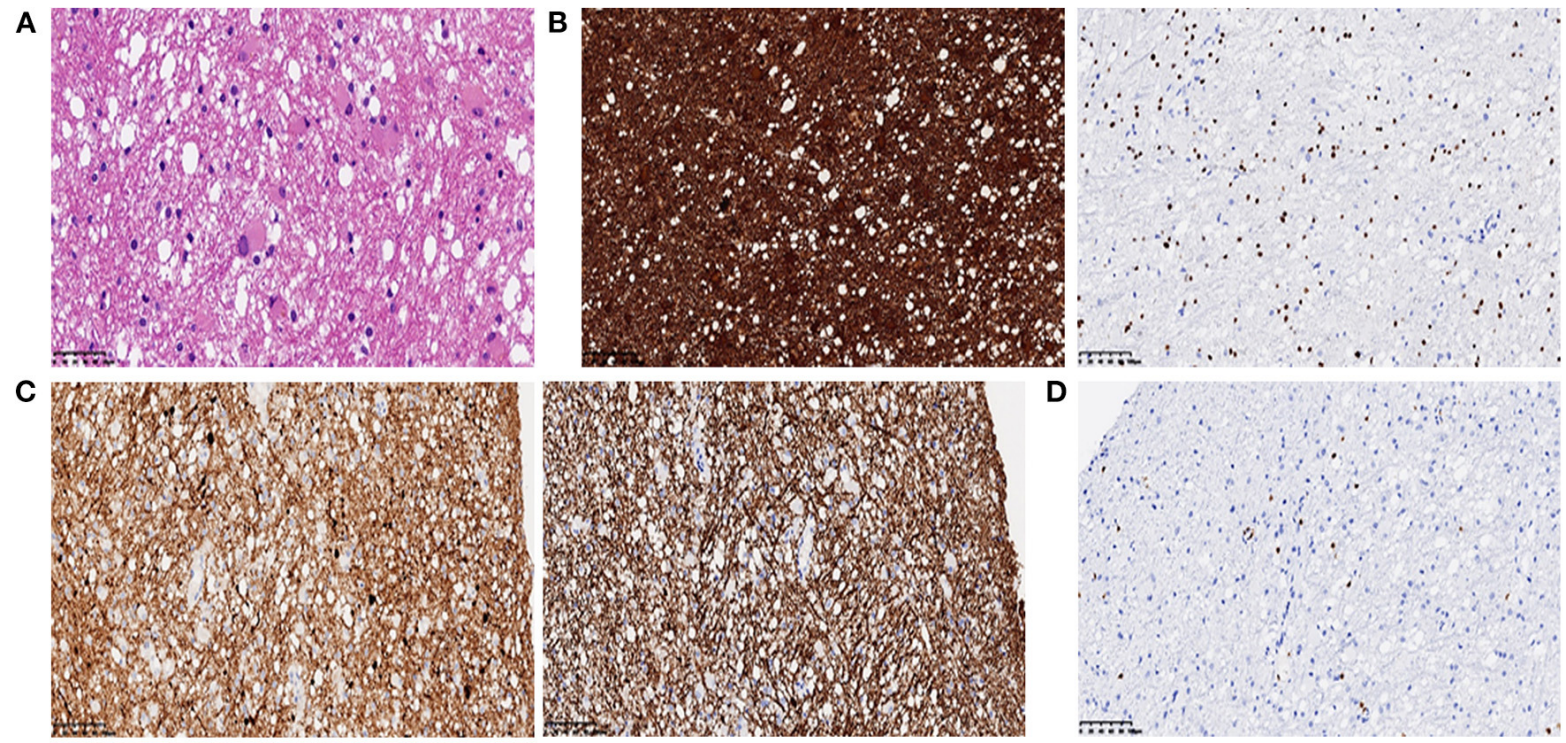
**(A)** Histopathology showed mild hydropic degeneration and gliocyte proliferation of the lesion (H & E, 400×); **(B)** GFAP (left panel) and Olig2 (right panel) immunohistochemistry showed positive staining of reactive gliocytes (200×); **(C)** SYN (left panel) and NF (right panel) immunohistochemistry showed positive staining of neurofilaments. **(D)** Lymphocytes highlighted by CD3 showed a perivascular infiltration pattern.

Brain magnetic resonance imaging (MRI) (Figure 1) demonstrated symmetric abnormalities on fluid-attenuated inversion recovery (FLAIR) hyperintensity within the white matter of the corona radiata, brachium pontis, and basal ganglia, with T1-weighted associated hypointensity (T1WI), without enhancing lesions but with correlating areas of hyperintensity on DWI. MR spectroscopy showed increased choline and a decreased N-acetyl aspartate (NAA) peak, and positron emission computed tomography (PET-CT) showed decreased fluorodeoxyglucose (FDG) uptake of the corona radiata, suggesting demyelination of the white matter. Accordingly, a stereotactic biopsy for corona radiata excluded the possibility of lymphomas. The pathology showed hydropic degeneration, gliocyte proliferation, and perivascular lymphocyte infiltration in the white matter (Figure 2).

He received methylprednisolone (500 mg over 4 days) and had transient cognitive improvement. Due to progressive confusion, low-dose methylprednisolone (80 mg) combined with immunoglobulin G (10 g), plasma exchange (one cycle) and rituximab (500 mg, four cycles) was administered. His consciousness improved, and he could respond with simple body language to the doctor's orders after therapy. However, he remained dysphagic, aphasic and disabled at the time of discharge. The SLE disease activity index (Table 1) was improved throughout the course of immunosuppressive therapy. Repeated MRI showed a stable lesion without new changes after 3 months (Figure 1).

At his one-year follow-up visit, he could perform oral intake and communicate verbally but had difficulty walking alone. Meanwhile, the disease remained stable, without recurrence. He was maintained on 40 mg of methylprednisolone daily. His rheumatological lab results had recovered to a normal status (Table 1). Subsequently, he was treated with rituximab (500 mg, 1 cycles, every year) to prevent relapse. Repeat brain MRI at 1 year showed minimal residual hyperintensity on DWI and a small resolution of T2/FLAIR abnormalities, such as in the brachium pontis (Figure 1).

### Literature Review

Twelve cases of leukoencephalopathy in NPSLE patients from 1991 to 2021 was searched by the online database PubMed utilizing the search strategies “NPSLE” and “leukoencephalopathy”. Further searches were undertaken to identify articles by searching string “white matter”, “intracranial hypertension”, and “cerebral oedema”. Additional cases not captured by the initial search method were found in the reference lists of identified cases. All cases that were selected presented leukoencephalopathy in the MRI. Data extracted from these case were age, sex, initial neurological symptoms, other previous organs involved, imaging findings-DWI, CSF, clinical treatment (outcomes), and follow up (Table 2). Our case has been rarely reported until now from the literature review. Most patient experienced aggressive treatment strategies earlier and had benign outcome.

**Table 2:** Summary of the clinical features of NPSLE patients with diffuse leukoencephalopathy.

**Patient (ref)**	**Age/Sex**	**Initial neurological symptoms**	**Other previous organs involved**	**Neuro-imaging (DWI)**	**CSF**	**Treatment (outcome)**	**Follow up**
1 (7)	38/F	Severe headache, syncope	Yes	Normal	Pressure ↑ chemistry: protein ↑ cytology: no cells OB(–)	IV steroid pulse (5 g over 3 days) –> 60 mg of oral prednisone and 200 mg of plaquenil daily (Marked improvement)	Relapse and death after one week
2 (8)	11/F	Generalized convulsions, prolonged unconsciousness	Yes	NA	Pressure: normal chemistry: protein 96 mg/dl ↑ cytology: no cells OB(–)	IV steroid pulse (1 g over 3 days) –>oral prednisolone (1 mg/kg) daily (Gradual improvement)	Stable rheumatological lab: normal MRI: T2 resolution (6 months later)
3 (9)	35/F	Headache, papilledema	Yes	NA	Pressure: 550 mmH_2_O ↑	–	–
4 (10)	32/F	Nausea, vomiting, diplopia	No	NA	–	IV MP, plasmapheresis and CTX (Death)	–
5 (11)	56/F	Gradual cognitive decline	Yes	NA	Chemistry: normal cytology: no cells OB(–)	80 mg of prednisone daily (Gradual improvement)	Stable MRI: T2 resolution (29 months later)
6 (12)	41/M	Headache, vertigo, papilledema	Yes	NA	Pressure: 350 mmH_2_O ↑ Pressure: 360 mmH_2_O ↑	IV MP (Marked improvement)	–
7 (13)	14/F	Headache, abducens palsy	Yes	NA	chemistry: normal cytology: no cells	IV steroid pulse (1 g over 3 days) –> tapered to 5 mg of prednisone and 200 mg of plaquenil daily (Gradual improvement)	Stable
8 (14)	33/F	Headache, nausea, vomiting,inattention	Yes	NA	Pressure: 240 mmH_2_O ↑	IV MP (1 g/day and tapered to 45 mg/day) (Marked improvement)	Relapse and death after two weeks
9 (15)	43/F	Gradual cognitive decline	Yes	NA	–	High doses of prednisolone (Gradual improvement)	–
10 (16)	13/F	Headache, blurry vision, neck pain	No	Normal	–	IV MP (1 g over 5 days), Rituximab and CTX –> 60 mg of prednisone daily and plaquenil (Marked improvement)	–
11 (17)	47/F	Headache	Yes	NA	Pressure: 250 mmH_2_O ↑ chemistry: protein 682 mg/dl ↑	IV steroid pulse (1 g over 3 days) –> tapered to 30 mg of prednisolone daily (Marked improvement)	Stable rheumatological lab: normal MRI: T2 resolution (1 year later)
12 (18)	19/F	Headaches, diplopia, papilledema	Yes	NA	cytology: 91 mm^3^ Pressure: 280 mmH_2_O ↑ chemistry: normal cytology: no cells	IV MP (1 g over 5 days) –> prednisone 1 mg/kg/d, plaquenil 400 mg/d and rituximab (500 mg every 6 months) (Marked improvement)	Stable MRI: T2 resolution (3 months later)

*Not available, NA; Methylprednisolone, MP; Cyclophosphamide, CTX; IV, Intravenous; OB, oligoclonal bands*.

## Discussion

This case report describes a male patient with a diagnosis of SLE with the initial and only onset of diffuse leukoencephalopathy on imaging, characterized symptomatically only by the rapid progression of cognitive dysfunction. He had a gradual, but incomplete, recovery with a stronger immunosuppression treatment. To the best of our knowledge, no case reports have been made associating these entities.

SLE affects women more frequently than men, with the preponderance of occurrence around childbearing age (15–44) (19). Similarly, 12 patients with leukoencephalopathy and NPSLE have been reported, largely involving women around a mean age of 34 years (Table 2). NPSLE is more frequent in the juvenile-onset SLE than in adults with SLE (20, 21). Moreover, nearly half of children with SLE will exhibit CNS involvement in the first year after initial diagnosis (20, 21). As shown in Table 2, three cases that were children has been reported with leukoencephalopathy as the first manifestation of juvenile-onset NPSLE. Additionally, men with SLE often have a more rapid progression in the clinical course with severe organ damage, resulting in a poorer prognosis than women (19).

Many patients already had a diagnosis of SLE and/or other systemic symptoms related to their disease prior to CNS involvement (22). In contrast, our patient made a diagnosis of SLE charactered by isolated CNS involvement. Lab testing reveals ANA at a titer of ≥1:80 and then SLE classification requires at least one clinical criterion and ≥10 points according to the 2019 EULAR/ACR criteria for SLE (23). Thus, our case shown ANA at a titer of 1:320, rapid progression of cognitive dysfunction (2 points), positive Coomb's test (4 points), an increase in anti-cardiolipin antibody IgM level (2 points), and low complement (C3 and C4) (4 points). On the above findings, a diagnosis of SLE was highly suspected. However, NPSLE was not specific, and a careful process of exclusion of causes was necessary. In the literature review about leukoencephalopathy, etiologies were warranted to be excluded, involving in metabolism, leukodystrophies and infection. Evidence suggests that SLE is strongly associated with B cell lymphoma (24, 25). In our case, an extensive workup was performed and these results were normal, including autoantibodies and sequencing of CSF, tandem mass spectrometry of blood and urine, exercise testing on serum lactate, cerebral MR spectroscopy, plasma ammonium, thyroid studies, folate, vitamin B12 and leukodystrophy-associated gene panel testing. Ultimately, our case ruled out a CNS infection, metabolism, tumors and genetics etc.

Most SLE patients with cognitive dysfunction have subtle or subclinical disease with a stable, improving or fluctuating course and rarely have a rapid progression to dementia (2). In addition, most SLE-related cognitive dysfunction occurs in the absence of active systemic lupus or major NPSLE events (2). MRI has shown lower hippocampal volumes in SLE patients with cognitive dysfunction than in those without cognitive dysfunction (26). Interestingly, our patient presented dementia with a rapid course as the initial manifestation of active SLE with diffuse leukoencephalopathy, unlike two patients who had gradual cognitive decline and other organs involved, as shown in Table 2. This characteristic is extremely rare and makes early diagnosis difficult.

Common MRI changes in NPSLE patients include small punctate focal lesions in periventricular and subcortical white matter hyperintensity, brain atrophy, and infarcts (27). Indeed, there have been case reports of patients with T2/FLAIR abnormalities with associated leukoencephalopathy, as shown in Table 2. However, we were unable to find an instance of a patient with a lasting DWI abnormality in leukoencephalopathy as the presenting sign of lupus upon initial diagnosis. Furthermore, patients have shown near-complete resolution of FLAIR abnormalities after immunosuppressive treatment. In contrast to these cases, our patient had a unique feature of hyperintense DWI and no reversible lesions at 3 months following a series of immunosuppressive therapies. There was a disappearance of hyperintensity on DWI after 1 year, while the FLAIR abnormalities remained. Hyperintensity on DWI MRI indicates that cytotoxic oedema has occurred and the damage is irreversible. This may be an indicator of severe NPSLE. What is more, inconsistent with most common findings, such as microinfarction and vasculitis in NPSLE (28), the pathology showed hydropic degeneration, gliocyte proliferation in our patient. Based on the relatively severe pathologic injury in brain, we might make an explanation on severe cognitive dysfunction in our patient.

Diagnosis of NPSLE is the lack of specific and sensitive CSF testing (6). As previous studies have shown, some autoantibodies have been suggested as a potential biomarker for diagnosis and therapeutic decision, such as antineuronal, anti-ribosomal P, and ant-NR2 antibodies (6). Autoantibodies may be detected in the CSF as a result of the transfer of peripherally produced autoantibodies across a breached blood brain barrier or increased intrathecal production (2). In addition, these autoantibodies could be synthesized intrathecally, supported by studies reporting that CSF lgG index/oligoclonal bands (OB) are frequently elevated in patients with NPSLE (29). Nevertheless, three NPSLE patients with leukoencephalopathy (Table 2), besides our case, presented negative, and these mechanisms will be warranted to further explore. Idiopathic intracranial hypertension (IIH), defined by an increased intracranial pressure without hydrocephalus or lesions on the MRI and with normal CSF composition, has been reported in a few patients with NPSLE (30). IIH usually indicates a favorable outcome (30). According to Table 2 findings, the increased intracranial pressure was a common manifestation in NPSLE patients with diffuse leukoencephalopathy. Therefore, difference from IIH, this subtype could be considered as vasogenic oedema and requires treatment aggressively to avoid death, especially combined immunotherapy with dehydrant usage, including acetazolamide, mannitol and furosemide.

High dose steroids have been a unifying treatment choice for neuropsychiatric lupus (31). In addition, due to patients' significant autoantibody loads and severe symptoms, rituximab and cyclophosphamide have been used to treat the severity of CNS involvement (32, 33). As Table 2 shows, most patients with acute SLE leukoencephalopathy had a full clinical recovery after alone high-dose steroid therapy. Of course, others had experienced incomplete or no clinical response and combined immunotherapy was warranted, including rituximab and cyclophosphamide. Overall, there have been reports that a large of patients experienced a gradual or marked improvement of clinical symptoms after treatment at period of acute course. In the follow-up visit, oral prednisone was used to maintain therapy and, whether examination or symptom, some patients were under a stable state. But, in the patients with lupus relapse, the aggressive immunosuppressants, such as rituximab, immunoglobulin G, plasmapheresis and even autologous stem cell transplantation, is warranted to avoid death as soon as possible (34). In our patient, given the low response to steroids, various methods were used for treatment, including immunoglobulin G, rituximab, and plasma exchange. Meanwhile, his SLE disease activity index recovered to baseline, and his orientation was improved (Table 1), confirming the response to treatment. At his one-year follow-up visit, his symptoms had improved further without recurrence following low-dose oral prednisone.

## Conclusion

NPSLE diagnosis is a challenge for clinicians, both at the diagnostic and therapeutic levels. Isolated leukoencephalopathy with hyperintense DWI on MRI in SLE, an indicator of severe NPSLE, has rarely been reported in the literature. Taken together, as a subtype of NPSLE, it is necessary to recognize severe NPSLE and provide aggressive immunosuppression therapy as soon as possible.

## Data Availability Statement

The datasets presented in this article are not readily available because of ethical and privacy restrictions. Requests to access the datasets should be directed to the corresponding author/s.

## Ethics Statement

The patients/participants provided their written informed consent to participate in this study. Written informed consent was obtained from the individual(s) for the publication of any potentially identifiable images or data included in this article.

## Author Contributions

YF, QX, and XY wrote the paper. TY implemented tissue staining. All authors read and approved the final manuscript.

## Conflict of Interest

The authors declare that the research was conducted in the absence of any commercial or financial relationships that could be construed as a potential conflict of interest.

## Publisher's Note

All claims expressed in this article are solely those of the authors and do not necessarily represent those of their affiliated organizations, or those of the publisher, the editors and the reviewers. Any product that may be evaluated in this article, or claim that may be made by its manufacturer, is not guaranteed or endorsed by the publisher.

## Abbreviations

SLE: Systemic lupus erythematosus
NPSLE: neuropsychiatric involvement in SLE
DWI: diffusion-weighted images
ESR: erythrocyte sedimentation rate
CRP: C-reactive protein
ANA: antinuclear antibody -speckle
CH50: total completement activity
CSF: Cerebral spinal fluid
MRI: Brain magnetic resonance imaging
FLAIR: fluid-attenuated inversion recovery
T1WI: T1-weighted associated hypointensity
NAA: N-acetyl aspartate
PET-CT: positron emission computed tomography
FDG: fluorodeoxyglucose.
